# Identifying health policy and systems research priorities for the sustainable development goals: social protection for health

**DOI:** 10.1186/s12939-018-0868-z

**Published:** 2018-09-27

**Authors:** Mary Qiu, Nasreen Jessani, Sara Bennett

**Affiliations:** 1 Johns Hopkins Bloomberg School of Public Health, 615 N Wolfe St, Baltimore, MD 21205 USA; 2 Washington, 20010 USA

**Keywords:** Health systems, Priority setting, Health policy, LMIC, Social protection, SDGs

## Abstract

**Background:**

There is an established body of evidence linking systems of social protection to health systems and health outcomes. The Sustainable Development Goals (SDGs) provide further emphasis on this linkage as necessary to achieving health and non-health goals. Existing literature on social protection and health has focused primarily on cash transfers. We sought to identify potential research priorities concerning social protection and health in low and middle-income countries, from multiple perspectives.

**Methods:**

Priority research questions were identified through two sources: 1) research reviews on social protection interventions and health, 2) interviews with 54 policy makers from Ministries of Health, multi-lateral or bilateral organizations, and NGOs. Data was collated and summarized using a framework analysis approach. The final refining and ranking of the questions was completed by researchers from around the globe through an online platform.

**Results:**

The overview of reviews identified 5 main categories of social protection interventions: cash transfers; financial incentives and other demand side financing interventions; food aid and nutritional interventions; parental leave; and livelihood/social welfare interventions. Policy-makers focused on the implementation and practice of social protection and health, how social protection programs could be integrated with other sectors, and how they should be monitored/evaluated. A collated list resulted in 31 priority research questions. Scale and sustainability of social protection programs ranked highest. The top 10 research questions focused heavily on design, implementation, and context, with a range of interventions that included cash transfers, social insurance, and labor market interventions.

**Conclusions:**

There is potentially a rich field of enquiry into the linkages between health systems and social protection programs, but research within this field has focused on a few relatively narrowly defined areas. The SDGs provide an impetus to the expansion of research of this nature, with priority setting exercises such as this helping to align funder investment with researcher effort and policy-maker evidence needs.

**Electronic supplementary material:**

The online version of this article (10.1186/s12939-018-0868-z) contains supplementary material, which is available to authorized users.

## Background

The 17 Sustainable Development Goals (SDGs) embody a systems-oriented approach to understanding the connections between human health, poverty, economic development and the environment. The first of the SDGs is to “End poverty in all its forms everywhere”. While there are a number of targets within this goal, a significant one (1.3) states that countries will *“Implement nationally appropriate social protection systems and measures for all, including floors, and by 2030 achieve substantial coverage of the poor and the vulnerable.”* At their simplest, systems of social protection exist to protect people against risks, particularly risks of falling into poverty, and to mitigate the effects of such risks when adverse events do occur.

### Understanding social protection and its linkages to health

The critical nature of linkages between social protection and health have long been recognized. For example, the 2008 World Health Organization Commission on the Social Determinants of Health presented, as one of its major recommendations the need to “improve living and working conditions and create social protection policy supportive of all” [1]. However the SDGs, and analyses of the SDGs have highlighted multiple linkages between different goals [2] and in particular, underscored the link between social protection and health [3]. The starting point for this work was the recognition of significant and extensive linkages between systems of social protection and health systems.

Social protection is often thought to encompass three main types of interventions: labor market interventions (such as regulations governing maternity leave and sickness leave); social insurance (including social health insurance, but also disability insurance and unemployment insurance) and diverse social safety nets that may include the provision of cash benefits, as well as food vouchers; and various services and programs including food for work and homeless shelters [4]. While social insurance programs are typically contributory (requiring beneficiaries to pay into the scheme in order to benefit from it), social safety net programs are non-contributory. Together, social protection schemes have been shown to: (1) Prevent risks ie. decrease the probability of an adverse event occurring (eg. labor laws that protect women’s rights to employment after giving birth [5]), (2) Mitigate risks ie. decrease the impact of the adverse event if it occurs (eg. by providing disability insurance, or workman’s insurance if people get injured at work [6]), and (3) Enable coping ie. relieve the impact of risk once it has occurred (eg. provide food aid to those below the poverty line [7]).

To-date, within the Health Policy and Systems Research (HPSR) field, there has been a considerable body of work addressing demand side strategies that bear some features of social protection, including cash transfers with a particular focus on conditional cash transfer (CCT) schemes [8–10]. There has also been substantial research addressing universal health coverage (target 8 under the health goal of the SDGs) including analyses of social health insurance schemes, user fee removal, and vouchers, but typically this research has focused relatively narrowly on programs to enhance access to health services, rather than considering more broadly the ties between health and social protection [11–13]. To-date the literature has examined, on the one hand, how broad social determinants – such as poverty, housing quality, employment etc. affect health, and on the other, how health systems can best be organized to protect and offer access to the poor, but there has been very little work that has investigated the linkages between health systems and systems of social protection.

There are at least four ways in which social protection systems affect health and health systems. First, social protection protects against risks that lead to financial and other vulnerabilities. The literature on social determinants of health has explored in some detail how structural factors such as employment and income, affect health status, and there is a well-established evidence base linking poverty with poorer health outcomes [14]. However, the linkages between social protection schemes and health have, with some notable exceptions been less frequently explored in low and middle income countries (LMICs) [15]. Second, social protection can facilitate and/or incentivize the utilization of health care services. Conditional cash transfer schemes may provide direct incentives for health service utilization, whereas unconditional cash transfers facilitate service utilization by helping to cover costs of transport, user fees etc. Third, social protection schemes may provide goods and services that are complementary to health care, such as providing food to patients on antiretrovirals, housing to people with mental health problems, or life skills for adolescents living with HIV. Finally, there may be practical or logistical linkages between health systems and social protection systems. For example, for particularly vulnerable members of society, case workers may play a role in coordinating their health care, housing and other social service needs, and health systems may rely upon social protection systems to identify the indigent who should receive health services free of charge.

### Aims

In recognition of the interconnectedness between health, and other social and environmental systems as highlighted by the SDGs, this study aimed to identify HPSR priorities for the SDGs in relation to social protection and health. In particular, we sought to identify knowledge gaps that currently exist for social protection and health, as perceived by both the research community and policy-makers, and to reconcile and prioritize these perspectives so as to develop a prioritized list of research questions within this domain.

This exercise sought to be internationally relevant, with a particular focus on LMICs. We chose to focus on LMICs given that to-date, the implementation of universal social protection mechanisms has taken place primarily in high-income settings. At the same time, there is a recognized need to expand universal social protection systems in such settings to protect the most vulnerable who face growing health insecurity, making this topic particularly relevant to health policy-makers [16]. The work reported here on social protection and health was one of three domains addressed by the over-arching exercise with the other domains addressed being multisectoral collaboration for health (Glandon et al., forthcoming), and participatory and accountable institutions (Scott et al., forthcoming).

## Methods

The methods draw upon previous Alliance for Health Policy and Systems Research (AHPSR) processes [17], and were adapted so as to broaden participation (via the internet) and accelerate the process.

The priority setting process involved three key steps: (1) an overview of reviews to identify what areas of social protection for health have been studied to date and what research questions have arisen as a result, (2) consultations with policy-makers across the WHO regions, particularly those from LMICs, to identify current priorities related to social protection for health in the context of the Sustainable Development Goals, and (3) an online interactive activity using the Co-Digital platform [18] where researchers from around the world were invited to refine and then rank the identified research priorities. Steps one and two were conducted concurrently, and the results were then integrated to create a list of initial research questions used in step 3. Final ranking of questions was then fed back to researchers for review and feedback.

### Overview of reviews

#### Search strategy

A Johns Hopkins University informationist collaborated with the research team to develop a search strategy for PubMed that was adapted for Embase, Scopus, PAIS International, Social Science Abstracts, PsycINFO, WHO Global Health Regional Indexes, and Ovid’s Global Health database. A combination of controlled vocabulary and keyword terms were used for each of the following concepts: (1) social* protection (2) health outcomes or access to health services (3) low and middle-income countries. Refer to Additional file 1 for full search terms.

The search results were limited to studies published between January 2000 and March 2017. Records from all databases were imported on February 24^th^, 2017. All duplicates were removed and unique citations were exported to Microsoft Excel for screening.

#### Study selection & criteria

Due to the large volume of articles initially retrieved, the study team decided to focus on reviews, excluding individual studies. MQ filtered the title and abstract columns of the database by searching for the word review. Two researchers (MQ and SB) independently reviewed the final database, using the following inclusion criteria:

- Published in English, Spanish, or Portuguese
- Both peer-reviewed and grey literature
- Includes at least one of the following: conditional/non-conditional transfers, social grants, non-monetary grants (such as food assistance), protective labor regulations, and includes programs delivered by sub-national or national level governments, rather than smaller scale, NGO run initiatives. Government run food assistance programs may encompass food stamps, food subsidies, women, infants and children feeding programs, and food price stabilization.
- Assesses one of the following: health impact, access to health services, effects on the health system, or the linkage between social protection and health systems
- Includes articles based on LMIC experience.

Titles and abstracts were then screened for full-text review. Studies were included if they met all inclusion criteria and were identified as being a review of any kind.

**Fig. 1:**
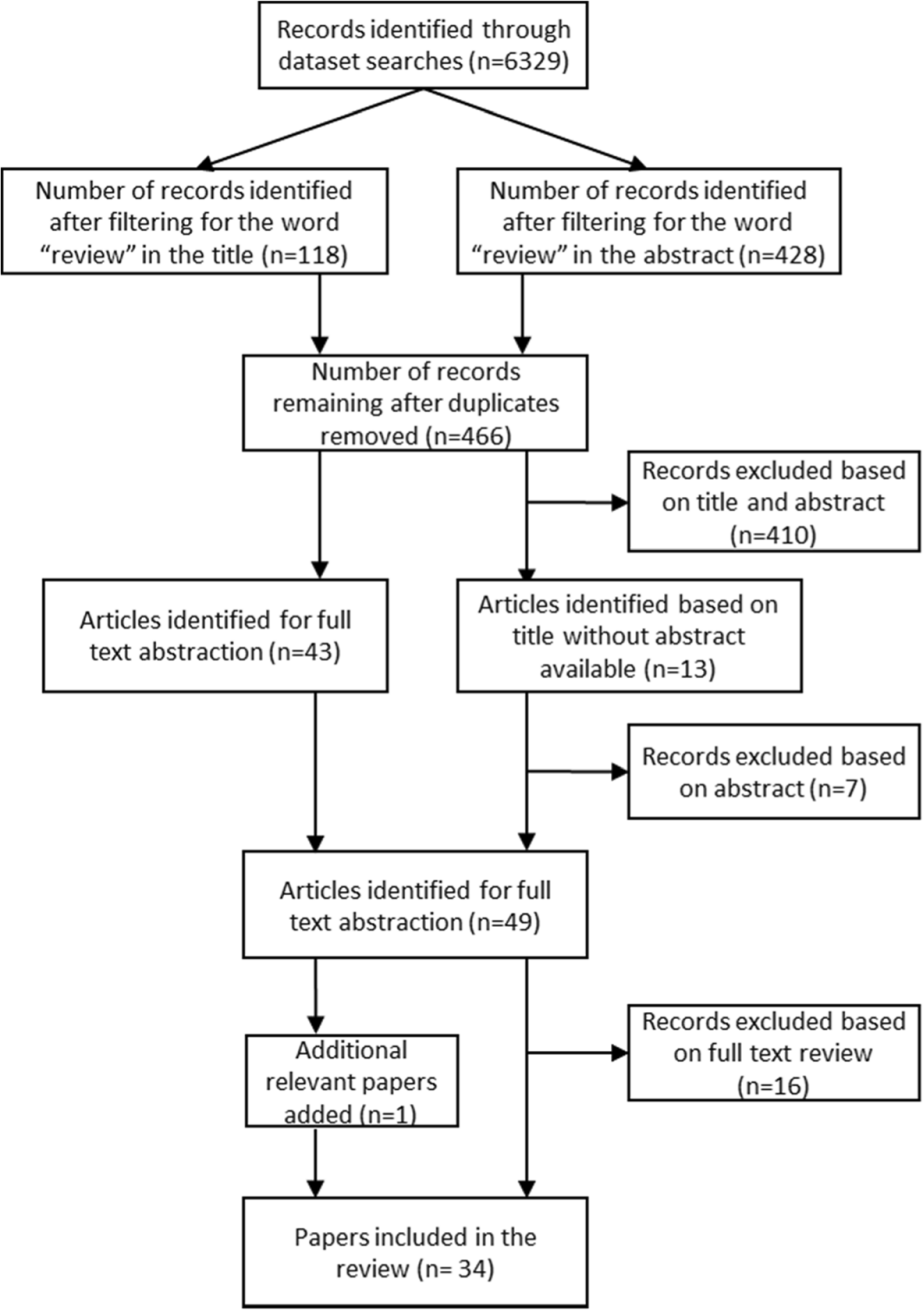
Diagram of search results for overview of reviews

The search resulted in a total of 6329 records. After filtering for reviews and removing duplicates, 466 articles remained for title and abstract screening. Of these, 49 were abstracted for full text review. During the review of the full text, 33 papers were identified as meeting inclusion criteria, and included for data abstraction, and one additional paper (referred to by one of the papers) was added for a total of 34 reviews (see Fig. 1). While the reviews were particularly rich in terms of information on Latin America, they provided information from LMICs in all six World Health Organization regions.

#### Data extraction

We created a Microsoft Office Excel based template to extract key information from each article, including: (1) publication details (authors, title, journal, year of publication, type of review), (2) study summary (types of interventions reviewed, target population, outcomes assessed), (3) details about each relevant intervention (description of intervention, impact, findings related to policy and implementation) and (4) the authors’ conclusions concerning the quality of research in this area, key research needs, and primary policy conclusions.

#### Quality assessment and data synthesis

While we did not exclude reviews due to quality considerations, the data extractor’s assessment of the quality of the review was included in the data extraction file.

### Policy-maker interviews

We targeted senior level policy-makers (typically directors and deputy directors, but including some secretaries, assistant secretaries and special advisors) within Ministries of Health or other relevant sectors such as Finance and Planning to participate. We also included senior staff from large multi-lateral or bilateral organizations to participate in the process. We leveraged two major global health conferences to recruit participants (Health Systems Global, Vancouver, Canada, November 2016, and the Prince Mahidol Awards Conference, Bangkok, Thailand, January 2017). In addition to this, study team members based in India, South Africa, Lebanon, and Argentina carried out consultations in their respective countries and regions with local policy-makers. While we did not target specific countries to include, we sought a balanced geographic representation across low and middle income countries. Participants were contacted via email, phone, or in-person. Some policy-makers were interviewed over the phone where an in-person interview was not possible, but the same interview protocols were followed.

Interviews began by providing some background information on the SDGs, and then requested respondents to reflect on health systems challenges that their country might face in meeting the SDGs, as well as any policy changes that were being considered in connection with the SDGs (without limiting the conversation to the three themes). The next set of questions focused on the three priority areas: (1) Protecting and promoting access to health services through systems of social protection (the focus of this paper), (2) Strengthening multi-sectoral collaborations for health, and (3) Developing more participatory and accountable institutions for health (2 and 3 are reported elsewhere). Within these themes, respondents were asked about particular challenges in these areas, proposed policy changes, and information and evidence needs. Participants were asked to respond to questions about the areas that they were most interested in which sometimes resulted in extensive discussions around one, two, or all three themes.

In total, we conducted 54 semi-structured interviews and two focus group discussions (FGD) across five WHO regions. Participants in 44 of the interviews and in both FGDs discussed the theme of social protection. Tables 1 and 2 present the breakdown of respondents by region.

**Table 1:** Respondent breakdowns by geographic region

WHO Region	Countries Included	Total No. Respondents Identified & Invited	Total No. Respondents Included	Total No. Respondents Discussing Social Protection	Interview Language(s)
Africa Region	Ghana, Kenya, Liberia, South Africa, Uganda, Zimbabwe	30	12	8	English
Region of the Americas:	Argentina, Caribbean Region (23 member states)	14	10	9	English, Spanish
South-East Asia Region:	Bhutan, India, Indonesia, Myanmar, Thailand	18	14	10	English
European Region		0	0	0	NA
Eastern Mediterranean Region	Bahrain, Jordan, Pakistan, Somalia, Tunisia	5	4	4	English, Arabic, French
Western Pacific Region	China, Kiribati, Laos, Philippines, Vanuatu	15	8	8	English, Mandarin
Multi/Bi-lateral Org/NGOs	NA	7	6	5	English

**Table 2:** Focus group discussion respondent breakdown

Focus Group Country	No. of Respondents Invited	Total No. Respondents Included	Focus Group Discussion Language
Bahrain	16	10	English
Jordan	17	17	Arabic

Conversations were recorded where possible, with detailed notes taken during the interview in lieu of verbatim transcriptions. We used a framework analysis approach [19], drawing upon the interview notes and recordings to populate a matrix for the various themes and ideas that came from the interviews.

#### Identification of research questions

We first synthesized and extracted the research needs identified from the overview of reviews. These were then collated and organized into broad themes. We then extracted research priorities from policy-maker interviews, categorizing them within our existing themes, or creating new ones where necessary. In many cases research questions included in the final listing were identified through both sources; However, in instances where questions were identified by only one source - either within the reviews, or by policy-makers, − we retained them as important and distinct topics that were not otherwise reflected in the existing list of questions. This led to a final set of 30 research questions for researcher prioritization.

### Consultation with researchers: Refining and ranking of research questions

In the final phase of the process we used both open solicitation and targeted invitations to identify researchers to assist with refining and ranking the final set of 30 questions. For the open solicitation process, we advertised the activity on the Health Systems Global webpage (www.healthsystemsglobal.org), and via Twitter. We received 127 responses, and of these, we included 72 respondents, purposively selected to represent low, middle, and high-income countries, and a diversity of regions and disciplinary backgrounds. An additional group of participants were targeted for inclusion based on their expertise in the field.

We used Co-Digital (www.codigital.com), an online platform, for this stage of the research prioritization process as this allowed for a large group to generate, refine, and prioritize ideas in a real time virtual environment. Participants received a draft of the report for the overview of reviews, and an excel spreadsheet depicting the source of each of the 30 research questions in advance of a two-stage “refine” and “rank” process.

From 4^th^–15^th^ September 2017, the first stage of the activity – “refinement” - permitted participants to suggest revisions to the original questions developed by the study team. Participants then voted on each edit, or “generation” of a question to determine which version moved forward for further refinement. Of the 72 individuals invited to respond, 30 participated, resulting in 207 distinct edits to the questions and 653 votes cast across all edits. Our team reviewed the final generation of each question, and incorporated edits so long as we felt that they did not change the original objective of the question. Where this happened, we reverted to earlier iterations of edits.

The finalized research questions were then fed back into Co-Digital for the second stage of the activity – “ranking” - where participants voted on priority research questions. The system used pairwise voting, where one question was ranked against one other. Participants received 20 votes to cast in total. We conducted this stage from the 21^st^- 27^th^ September. In this stage, 32 individuals participated, generating 620 votes. Instructions to participants asked them to consider the potential impact that research on a question might have, as well as tractability, and the extent to which answering the question would benefit poor and marginalized communities.

Final ranking of the questions was calculated based on the total number of times each question “won” in a vote, out of the total number of times the question was included in a vote. For example, if a question appeared in 20 votes, and “won” 17 of them, then they received a final score of 85%.

Final results were shared with participants and we solicited feedback on the overall process and end result.

## Results

### Overview of reviews

#### Categories of social protection interventions

Many of the reviews addressed multiple different types of social protection mechanisms, with a focus on cash transfers being by far the most common. Of the 34 reviews that met all inclusion criteria, the majority (31/34) included or focused on cash transfers (unconditional and/or conditional). Four reviews focused on or included a component on food aid (of which two included cash transfers as a food aid intervention), and one on paid maternity leave. While cash transfer programs (particularly conditional cash transfer programs) dominated the literature, researchers sometimes also included components examining specific social protection initiatives such as services to link vulnerable households to other social welfare programs, or interventions related to improving livelihoods. Where reviews included a range of financial incentive programs or demand-side interventions, we focused solely on those that could be defined as “social protection” using the descriptions provided above. Accordingly, interventions such as the provision of vouchers, or user fee removals were excluded from the analysis. Table 3 lists all types of interventions included and the corresponding review papers.

**Table 3:** Type of interventions included in overview of reviews and the corresponding articles

Intervention	Review Papers
Cash Transfers (Conditional & Unconditional)	[8–10, 22, 23, 26–28, 30–49]
Other Financial Incentives/Demand Side Financing Interventions	[23, 26, 28, 31, 32, 37, 38, 48, 50–52]
Food Aid and Nutritional Interventions	[21, 22, 24, 34]
Parental Leave	[25]
Livelihood or Other Types of Social Welfare	[21, 34, 42]

#### Questions for future research identified in review papers

Within the social protection and health literature, work on CCTs was by far the most substantive, but even in this domain many review authors underscored the significant limitations of current knowledge. CCTs differ extensively, in terms of their target audience, the types of behaviors they are seeking to change, the nature of conditions imposed, and the level and frequency of payments. So, while there is considerable evidence to suggest that CCTs can be effective in changing behaviors, many questions remain about how such programs should be designed, implemented, and tailored to local conditions. Further, important questions about how CCTs compare to unconditional cash transfer programs, and how any type of cash transfer may be combined with other types of intervention such as care, counselling or linkages to other services, and the combined effects of such packages of interventions were raised. Several of the reviews (as well as the policy-maker interviews) noted potential concerns about how cash transfers may distort incentives and negatively affect “risky” behavior (for example how paid maternity leave may encourage women to have more children). While many studies have already demonstrated that such perverse effects rarely occur, it is possible that additional work of this nature is needed in order to garner stronger political support for social protection programs as well as to improve program design.

Many of the papers included in the overview of reviews reviewed interventions concerned with maternal, neonatal and child health; nutrition; and sexual health including HIV, with single reviews looking at mental health, non-communicable diseases (NCDs) (as one amongst several health conditions), and disability. In contrast to the Millennium Development Goals (MDGs), the SDGs have explicit targets addressing NCDs, mental health, substance abuse and injuries due to road traffic accidents. Strengthening linkages between health and social protection systems may be particularly important to address these emerging health priorities. For example, CCTs may be effective strategies for shifting individual behaviors in ways that help people to avoid NCDs [20]. For those already suffering from long term illness or disability associated with NCDs, mental health conditions or injuries, measures to protect their incomes, and connect them with other social services may be particularly critical.

### Policy-maker interviews

#### Questions for future research identified from policy-maker consultations

Policy-makers spoke to a range of issues related to social protection initiatives and health that they felt warranted further investigation, many of which focused on the practicality and “how to” of social protection programs. In particular, policy-makers raised questions around eligibility, fraud and abuse within social protection programs, and avoiding dependencies amongst recipients. For example, policy-makers in Indonesia, Ghana, Laos, India, and the Bahrain asked what the best way of determining eligibility would be to ensure that the most-needy are targeted. Regarding fraud and abuse, both policy-makers in the Philippines and South Africa described the issue of gaming or double-dipping across the system, and asked how this might be prevented. In Bahrain and Jordan, policy-makers expressed concern around overuse of the system, and how to monitor appropriate use of social protection programs. And in South Africa and Jordan, policy-makers wanted to know how to minimize dependency and couple skills development with social protection so that beneficiaries can be graduated.

Respondents also wondered about how social protection programs can be best adapted to their own unique contexts, given that the bulk of research on large-scale social protection programs has taken place in Latin America. For example, a policy-maker from Somalia wanted to know how social protection programs could be best adapted to fragile state and conflict settings. In Vanuatu and China, policy-makers wondered how lessons learned in other countries could be used to design programs that work in their settings.

Another area of research frequently mentioned was that of integrating social protection programs for health with other sectors, such as education. Respondents ranging from Laos, South Africa, India, Indonesia, Argentina, and Kiribati asked questions related to both how programs can be integrated for efficiency, as well as how health programs may impact non-health sectors, and vice versa.

Finally, the topic of monitoring and routine data systems was brought up across multiple interviews, as policy-makers wanted to know how they could ensure that investments made are having the right results.

### Priority research questions

The table in Additional file 2 lists the finalized 31 priority research questions that were the result of the refining and ranking exercise, while Additional file 3 depicts the original research questions and their source questions from the review and consultations. Research questions can be broadly characterized as those investigating broad implementation issues around social protection programs (including the mitigation of corruption and dependency), the effects of social protection programs and the pathways through which these effects can occur, how specific types of social protection programs compare to other types of interventions, and how social protection programs can work for different populations (such as migrant workers and refugees). Several of the questions (#1,2,4,10,11,12,15,29,30) center around cash transfer programs, as this was the most commonly described social protection program in the literature, however not all of these questions had substantial support from policy-makers. One additional question (#31) was added as a consequence of the suggested refinements made by participants.

#### Final research questions & ranking

Table 4 presents the final list of 31 questions, categorized by research need. Table 5 presents the top ten questions that emerged from the ranking activity with their final score. The top two questions in particular, appeared very popular, with relatively high final scores. The top ranked three priority questions all had strong support from both the literature and policy-makers, with the third ranked question (concerning integration of initiative across sectors) being of particular interest to policy-makers. By contrast, the fourth and sixth ranked question were derived mainly from the literature and had limited policy-maker support. Table 6 displays the top five most highly cited questions by source (based on a count of how many times they appeared), alongside the top five questions from the final ranking.

**Table 4:** Finalized questions by theme

Global Theme	Research Need	Finalized Research Quesiton
Evidence of Effectiveness	Increase overall evidence base for demand side interventions on impact	What are the effects of conditional cash transfer programs on healthcare quality, coverage and outcomes across settings in low and middle income countries?
		What are the effects of unconditional cash transfer programs on healthcare quality, coverage and outcomes across settings in low and middle income countries?
		How do the characteristics of cash transfers such as the amount, frequency, method of payment, etc., affect intended outcomes, particularly enrollment of the target population?
		What are the impacts of social protection programs in conflict affected settings and their effectiveness in improving health outcomes and access to health services?
	Longevity of effects	What are the long-term effects of cash transfer programs or social protection programs on behavior change, and how sustainable have they been?
		How can demand-side financing for health be made financially sustain able?
		How can social protection programs for health be designed, implemented, and evaluated to ensure sustainability and scalability in low and middle income countries, including conflict affected settings?
	Increase understanding of the equity effects of CCTs/demand side financing	What is the impact of social protection initiatives on health equity outcomes and equitable access to quality health care services for poor and marginalized populations?
Evidence of Effectiveness for Specific Services	Increasing evidence around the way food aid is delivered and its effect on intended outcomes	How do the characteristics of food aid programs (e.g. source, amount, frequency, mode, recipient etc.) affect intended health-related outcomes (e.g. morbidity, sustained behavior changes, drug adherence, labor market participation etc.)?
	Increasing evidence for paid maternity leave and the link to health outcomes	What are the impacts of different forms of maternity leave (paid vs. unpaid, length, etc.) on maternal and child health outcomes?
Evidence of Effectiveness for Cost Effectiveness	Increasing evidence of DSF on OOP	How do demand-side financing or CCT programs affect out-of-pocket spending on health?
	Increasing evidence on cost-effectiveness and efficiency	How cost-effective are CCT programs compared to supply-side interventions (e.g. strengthening quality of infrastructure and expanding services) in improving health?
		How cost-effective are CCT programs compared with other types of demand side interventions (e.g. UCTs, vouchers, behavior change, communication) in improving health coverage and health outcomes?
Pathways of impact/methods	Strengthen our understanding in the ways that internal and external factors influence SP programs and their effectiveness	What are the pathways through which social protection programs affect clinical and nonclinical outcomes, and what are the implications on program design?
		What are the contextual factors that influence the effectiveness of conditional and unconditional cash transfer schemes for health?
	Strengthen our understanding in the ways that SP programs affect intergenerational relationships	How do social protection programs (e.g. cash transfers) affect intergenerational and gender relationships at the community and household level?
		How do social protection programs for health affect intergenerational poverty and social mobility?
	Strengthen our understanding of the importance of conditionality for cash transfer programs	How does conditionality in cash transfer programs affect behavioral changes for disease prevention and treatment?
Unintended Consequences	Strengthen our understanding of the unintended consequences of social protection programs	What are the unintended health-related consequences of social protection programs?
		How can social protection programs be designed to minimize dependency and promote productivity amongst beneficiaries?
		What is the extent of fraud and abuse in health-related social protection programs, and how can social protection programs be designed to show accountability?
Program Implementation	Identify tools to help with determining eligibility for SP interventions.	What tools and systems can be used to assess and apply eligibility criteria for health-related social protection programs?
	Strengthen our understanding on how SP programs should best integrate or interact with specific groups of people or other sectors	How can the community/civil society be engaged to help design, implement and evaluate social protection programs?
		How can various social protection initiatives be best integrated or harmonized across sectors?
		How do social protection programs influence the interaction between public and private health care providers with regards to service availability, quality of care and utilization?
		How do we provide social protection programs to refugee populations without undermining support for nationals?
		How can informal sector and migrant workers be effectively covered by health-related social protection systems?
		How can social protection schemes help in ensuring that the most vulnerable such as the disabled are provided with people-centered and integrated services?
		How can social protection systems help in protecting people from domestic violence and its consequences?
	Strengthen our understanding of linkages between SP for health and governance	How do social protection programs contribute to state building?
Data reliability and validity	Strengthen our understanding on how SP programs can be best monitored	How can routine information systems be strengthened and used to monitor and evaluate social protection systems for health?

**Table 5:** Ten highest ranked research questions

Rank	Question	Final Score (% of times question was preferred over the alternative)
1	How can social protection programs for health be designed, implemented, and evaluated to ensure sustainability and scalability in low and middle-income countries, including conflict affected settings? (#6)	80.5%
2	What are the contextual factors that influence the effectiveness of conditional and unconditional cash transfer schemes for health? (#29)	71.1%
3	How can various social protection initiatives be best integrated or harmonized across sectors? (#21)	66.7%
4	How cost-effective are CCT programs compared to supply-side interventions (e.g. strengthening quality of infrastructure and expanding services) in improving health? (#11)	65.9%
5	What are the impacts of social protection programs in conflict affected settings and their effectiveness in improving health outcomes and access to health services? (#3)	63.6%
6	What are the pathways through which social protection programs affect clinical and nonclinical outcomes, and what are the implications on program design? (#13)	61.8%
7	What is the impact of social protection initiatives on health equity outcomes and equitable access to quality health care services for poor and marginalized populations? (#7)	61.4%
8	How can routine information systems be strengthened and used to monitor and evaluate social protection systems for health? (#25)	57.5%
9	How do social protection programs influence the interaction between public and private health care providers with regards to service availability, quality of care and utilization? (#22)	55.6%
10	What are the effects of unconditional cash transfer programs on healthcare quality, coverage and outcomes across settings in low and middle-income countries? (#30)	55.0%

**Table 6:** Comparison of most cited and most highly ranked questions by activity

Rank	Most Cited: Scoping Review (count)	Rank	Most Cited: Policy-Maker Interviews (count)	Rank	Highest ranked (% votes)
1	Q13: What are the pathways through which social protection programs affect clinical and nonclinical outcomes, and what are the implications on program design? [13]	1	Q25: How can routine information systems be strengthened and used to monitor and evaluate social protection systems for health? [13]	1	Q6: How can social protection programs for health be designed, implemented, and evaluated to ensure sustainability and scalability in low and middle-income countries, including conflict affected settings? (80.5%)
2	Q1: What are the effects of conditional cash transfer programs on healthcare quality, coverage and outcomes across settings in low and middle-income countries? [11]	2	Q21: How can various social protection initiatvies be best inegrated or harmonized across sectors? [12]	2	Q29: What are the contextual factors that influence the effectiveness of conditional and unconditional cash transfer schemes for health? (71.1%)
3	Q30: What are the effects of unconditional cash transfer programs on healthcare quality, coverage and outcomes across settings in low and middle-income countries? [9]	3	Q19: What tools and systems can be used to assess and apply eligibility criteria for health-related social protection programs? [8]	3	Q21: How can various social protection initiatives be best integrated or harmonized across sectors? (66.7%)
3	Q12: How cost-effective are CCT programs compared with other types of demand side interventions (e.g. UCTs, vouchers, behavior change, communication) in improving health coverage and health outcomes? [9]	4	Q18: What is the extent of fraud and abuse in health-related social protection programs, and how can social protection programs be designed to show accountability? [6]	4	Q11: How cost-effective are CCT programs compared to supply-side interventions (e.g. strengthening quality of infrastructure and expanding services) in improving health? (65.9%)
4	Q8: How do the characteristics of food aid programs (e.g. source, amount, frequency, mode, recipient etc.) affect intended health-related outcomes (e.g. morbidity, sustained behavior changes, drug adherence, labor market participation etc.)? [6]	5	Q4: What are the long-term effects of cash transfer programs or social protection programs on behavior change, and how sustainable have they been? [5]	5	Q3: What are the impacts of social protection programs in conflict affected settings and their effectiveness in improving health outcomes and access to health services? (63.6%)
		5	Q26: How can informal sector and migrant workers be effectively covered by health-related social protection systems? [5]		

## Discussion

Based upon both the available literature reviews and semi-structured interviews with diverse policy-makers, this study identified a list of 31 priority health policy and systems research questions concerning social protection and health. This list of questions was then refined and ranked so as to identify the highest priority research questions. The highest ranked ten questions identify quite distinct issues, and span the gamut from very process oriented questions (for example concerning how to implement social protection schemes) through to questions of impact and effectiveness. While there is a mix of questions in the top ten, it appears that the more highly ranked questions concern issues of design, implementation, and the importance of context. The most highly ranked question, question number 6, also emphasizes the importance of scalability and sustainability, suggesting a need to move away from relatively small-scale research-based projects towards larger national initiatives. There is also a notable interest in questions regarding the role of social protection in protecting health in conflict-affected settings.

The diversity across the final 31 questions is likely reflective of the overall lack of evidence around social protection mechanisms and health, in addition to the fact that social protection mechanisms can take place in many different forms for a multitude of populations, few of which have been tested at scale. From Table 6, it can be seen that those questions most highly ranked by researchers were generally not the most cited in the reviews or by policy-makers, with the exception of question 21 which was frequently mentioned by policy-makers and ranked number 3 in the final exercise. Given the focus in the existing HPSR literature on CCTs, it is hardly surprising that the most commonly cited questions in the reviews addressed different aspects of CCTs, or how CCTs compared to alternative strategies (such as unconditional cash transfers and supply side interventions. However, such questions did not feature at all in the questions most frequently cited by policy-makers, and were also less prominent in the final ranking exercise carried out by researchers, indicating potential divergences in perceived need. CCTs may be more important in some regions than others –much of the current literature is informed by Latin American experience– and policy-makers from that region may value research in this field more. In general, policy makers appear to value more practice oriented questions, focusing on the ‘how-to’, while researchers are more interested in how SP interventions have effect, and to what degree. But, overall it appears that HPSR researchers (and their funders) need to adopt a broader perspective in thinking about how health intersects with social protection, and the distinct pathways that social protection can take to improve health.

In drawing upon the overview of reviews and the policy-maker interviews to identify research questions to be used in the ranking exercise, it was relatively rare that the research team framed a question around a specific social protection modality. However, both the literature reviews and the interviews identified a range of social protection interventions with relevance to health. For example, questions regarding food aid and its effects on health were commonly posed in the reviews examined, and policy-makers expressed interest in better understanding the effects of maternity benefits, sickness benefits, and pension schemes among other forms of social protection [21, 22]. Some policy-makers framed their interests in terms of specific populations – notably the most vulnerable, disabled, children, refugees, migrant workers, the elderly, and those suffering from domestic violence. Policy-makers and the literature reviews also identified several important distributional questions, including inter-generational distribution, as well as intra-household distribution and gendered effects of social protection [23, 24]. Some researchers focused their reviews on specific disease or health conditions (such as TB, HIV/AIDS, maternal health) offering another lens through which to approach such research [9, 23, 25–28]. Overall, the field of social protection and health offers a rich, and relatively sparsely touched field for research. Many of the potential research questions identified will require health policy and systems researchers to collaborate with those in distinct fields, notably researchers working directly on social protection systems, but also those with interests in labor markets, poverty and equity.

While the priority setting process described here has a number of strengths, particularly in terms of being multi-faceted and engaging both researchers and research users, it also has a number of limitations. First, although participants in the ranking exercise were invited to reflect on the overall process and the ranked list of priorities that emerged, very few chose to do so, perhaps indicating respondent fatigue after what had been a relatively extensive engagement process. Second, due to the difficulty in eliciting research questions from policy-makers, who are at ease articulating problems or policy concerns, but are unfamiliar with research [29], this study did not directly ask policy-makers about research questions, but rather focused on understanding the problems they faced, the types of policies they were considering, and their evidence needs. Translating these perspectives into research questions was inevitably somewhat subjective in nature.

Thirdly, a number of interviews were conducted by phone rather than in-person, due to travel, time, and financial constraints. In such cases, we attempted to minimize potential biases by using the same interview guide and introductions across all respondents, however, we recognize that different modalities of communication may elicit somewhat different interpretations of questions and responses. We attempted to minimize such biases by limiting phone interviews to the greatest extent possible.

Fourthly, we recognize that FGD responses may have been influenced as a result of groupthink, in comparison to individual interviews. In such instances, we feel that the face-to-face nature of these interactions allowed the research team to minimize misinterpretation, and for respondents to seek clarity where questions were unclear.

Finally, in terms of limitations, this exercise was designed to inform global level stakeholders rather than reflect the specific research and evidence needs of any given country. Unfortunately, it continues to be the case that very few countries have articulated their own research priorities. The Council on Health Research for Development website (https://www.healthresearchweb.org/en/national_priorities_for_health_research) identifies nearly 40 countries which have some kind of health research priority document, but these often appear to be outdated (10 or 15 years old), or focused on some specific aspect of health (such as HIV/AIDS). The research priorities here may help inform country-level dialogue about health policy and systems research priorities, but should not be seen as replacing the need for local priority setting.

## Conclusion

There is potentially a rich field of enquiry into the linkages between health systems and social protection programs, but to-date research within this field has focused on a few relatively narrowly defined areas. The SDGs provide a spur to the expansion of research of this nature, however the final measure of success in a research priority setting exercise is the extent to which it informs research conducted, funded and used. Our aim in conducting this work was to better align research funder investment, researcher effort, and policy-maker evidence needs. We hope that this paper will inform future calls for research and will generate new evidence in social protection interventions that have previously been neglected. Finally, we hope that such efforts will lead to strengthened systems of social protection for health, as well as greater synergies between health systems and social protection schemes.

## Additional files

Additional file 1: Search terms applied to the overview of reviews. (DOCX 18 kb)

Additional file 2: Final research questions with original sources. (DOCX 90 kb)

Additional file 3: Original research questions and their source questions. (XLSX 28 kb)

## Abbreviations

AHPSR: Alliance for health policy and systems research
CCT: Conditional cash transfers
FGD: Focus group discussion
LMIC: Low and middle-income countries
MDGs: Millennium development goals
NCDs: Non-communicable diseases
SDGs: Sustainable development goals
