# Supplementary material for: Identifying health policy and systems research priorities for the sustainable development goals: social protection for health
Source: Int J Equity Health. 2018 Sep 27;17:155. doi: 10.1186/s12939-018-0868-z (PMC6161373; doi:10.1186/s12939-018-0868-z)
Supplement: Supplementary file 1 — Search terms applied to the overview of reviews. (DOCX 18 kb) [file 12939_2018_868_MOESM1_ESM.docx]

**Additional file 1**: Search Terms for Overview of Reviews

PubMed Search

Feb 13 2017

Limits: 2000-2017

1372 records

_____________________________________________________________________________________

**(#1 OR #2) AND (#3 OR #4) AND (LMIC concept)**

**_____________________________________________________________________________________**

#1 "Reimbursement, Incentive"[Mesh] OR "Food Assistance"[Mesh] OR "Parental Leave"[Mesh] OR "Pensions"[Mesh]

OR

#2 "social protection"[tw] OR "cash transfers"[tiab] OR "cash transfer"[tiab] OR "cash in kind"[tiab] OR "financial incentives"[tiab] OR "financial incentive"[tiab] OR "Incentive Reimbursements"[tw] OR "Incentive Reimbursement"[tw] OR "Reimbursement Incentive"[tw] OR "Reimbursement Incentives"[tw] OR "monetary incentive"[tiab] OR "monetary incentives"[tiab] OR "social assistance"[tiab] OR vouchers[tiab] OR "social insurance"[tiab] OR "social grants"[tiab] OR "child assistance grants"[tiab] OR "Child grants"[tiab] OR "disability grants"[tiab] OR "cash grants"[tiab] OR "food assistance"[tiab] OR "food aid" OR "food subsidies"[tiab] OR "supplemental nutrition assistance program"[tiab] OR "food stamp"[tiab] OR "food stamps" OR "free schooling"[tiab] OR "free schools"[tiab] OR "labor regulations"[tiab] OR "unemployment insurance"[tiab] OR "maternity leave"[tiab] OR "paternity leave"[tiab] OR "parental leave"[tiab] OR "minimum wage"[tiab] OR pensions[tiab] OR pension[tiab] OR "disability benefits"[tiab] OR "disability grant"[tiab] OR "disability grants"[tiab] OR "unemployment benefits"[tiab] OR "child support"[tiab] OR "parental support"[tiab] OR "employment protection"[tiab]

AND

#3 "quality of life"[mesh] OR "preventative health services"[mesh] OR "health behavior"[mesh] OR "Poverty"[Mesh] OR "Birth Weight"[Mesh] OR "Infant, Extremely Low Birth Weight"[Mesh] OR "Infant, Very Low Birth Weight"[Mesh] OR "Malnutrition"[Mesh]

#4 "health systems"[tiab] OR "Social health insurance"[tiab] OR "social insurance"[tiab] OR "Health services"[tiab] OR "health service"[tiab] OR "universal health care"[tiab] OR "national insurance schemes"[tiab] OR "National Health Programs"[tiab] OR "National Health Insurance"[tiab] OR "social security"[tiab] OR illness[tiab] OR "health care services"[tiab] OR "healthcare services"[tiab] OR "healthcare service"[tiab] OR "health outcomes"[tiab] OR "health status"[tiab] OR "child health"[tiab] OR "quality of life"[tiab] OR "life quality"[tiab] OR "health promotion"[tiab] OR "social welfare" OR "Child Welfare"[tiab] OR "Child Well Being"[tiab] OR "adolescent well being"[tiab] OR "adolescent welfare"[tiab] OR "health outcomes"[tiab] OR poverty[tiab] OR "impoverishment"[tiab] OR "Birth Weight"[tiab] OR "malnutrition"[tiab] OR "nutritional deficiency"[tiab] OR "nutritional deficiencies"[tiab] OR "Nutritional outcomes"[tiab] OR "Nutritional outcome"[tiab] OR "height for age"[tiab] OR "weight for age"[tiab] OR "ponderal"[tiab] OR "anthropometric"[tiab] OR "fully immunized"[tiab] OR

(("food adherence"[tiab] OR "food assistance"[tiab] OR "food security"[tiab]) AND ("treatment"[tiab])) OR ((maternal[tiab] OR adolescent[tiab] OR teen[tiab] OR teens[tiab] OR teenager[tiab] OR teenagers[tiab] OR "Child, Preschool"[Mesh] OR child[tiab] OR children[tiab] OR “pre-schooler"[tiab] OR “preschooler"[tiab] OR “under 5"[tiab] OR “under five"[tiab] OR “under fives"[tiab] OR "less than five"[tiab] OR "childhood"[tiab] OR toddler*[tiab] OR "young patient"[all fields] OR "young patients"[tiab] OR "boy"[tiab] OR "boys"[tiab] OR girl*[tiab] OR "young age"[tiab]) AND (prevention[tiab] OR "mortality"[tiab] OR "death"[tiab] OR "deaths"[tiab]))

LMIC

<https://datahelpdesk.worldbank.org/knowledgebase/articles/906519-world-bank-country-and-lending-groups>

((“emerging country”[all fields] OR “emerging countries”[all fields] OR “emerging nation”[all fields] OR “emerging nations”[all fields] OR “emerging population”[all fields] OR “emerging populations”[all fields] OR "developing country"[tiab] OR "developing countries"[tiab] OR "developing nation"[tiab] OR "developing nations"[tiab] OR "developing population"[tiab] OR "developing populations"[tiab] OR "developing world"[tiab] OR "less developed country"[tiab] OR "less developed countries"[tiab] OR "less developed nation"[tiab] OR "less developed nations"[tiab] OR "less developed population"[tiab] OR "less developed populations"[tiab] OR "less developed world"[tiab] OR "lesser developed country"[tiab] OR "lesser developed countries"[tiab] OR "lesser developed nation"[tiab] OR "lesser developed nations"[tiab] OR "lesser developed population"[tiab] OR "lesser developed populations"[tiab] OR "lesser developed world"[tiab] OR "under developed country"[tiab] OR "under developed countries"[tiab] OR "under developed nation"[tiab] OR "under developed nations"[tiab] OR "under developed population"[tiab] OR "under developed populations"[tiab] OR "under developed world"[tiab] OR "underdeveloped country"[tiab] OR "underdeveloped countries"[tiab] OR "underdeveloped nation"[tiab] OR "underdeveloped nations"[tiab] OR "underdeveloped population"[tiab] OR "underdeveloped populations"[tiab] OR "underdeveloped world"[tiab] OR "middle income country"[tiab] OR "middle income countries"[tiab] OR "middle income nation"[tiab] OR "middle income nations"[tiab] OR "middle income population"[tiab] OR "middle income populations"[tiab] OR "low income country"[tiab] OR "low income countries"[tiab] OR "low income nation"[tiab] OR "low income nations"[tiab] OR "low income population"[tiab] OR "low income populations"[tiab] OR "lower income country"[tiab] OR "lower income countries"[tiab] OR "lower income nation"[tiab] OR "lower income nations"[tiab] OR "lower income population"[tiab] OR "lower income populations"[tiab] OR "underserved country"[tiab] OR "underserved countries"[tiab] OR "underserved nation"[tiab] OR "underserved nations"[tiab] OR "underserved population"[tiab] OR "underserved populations"[tiab] OR "underserved world"[tiab] OR "under served country"[tiab] OR "under served countries"[tiab] OR "under served nation"[tiab] OR "under served nations"[tiab] OR "under served population"[tiab] OR "under served populations"[tiab] OR "under served world"[tiab] OR "deprived country"[tiab] OR "deprived countries"[tiab] OR "deprived nation"[tiab] OR "deprived nations"[tiab] OR "deprived population"[tiab] OR "deprived populations"[tiab] OR "deprived world"[tiab] OR "poor country"[tiab] OR "poor countries"[tiab] OR "poor nation"[tiab] OR "poor nations"[tiab] OR "poor population"[tiab] OR "poor populations"[tiab] OR "poor world"[tiab] OR "poorer country"[tiab] OR "poorer countries"[tiab] OR "poorer nation"[tiab] OR "poorer nations"[tiab] OR "poorer population"[tiab] OR "poorer populations"[tiab] OR "poorer world"[tiab] OR "developing economy"[tiab] OR "developing economies"[tiab] OR "less developed economy"[tiab] OR "less developed economies"[tiab] OR "lesser developed economy"[tiab] OR "lesser developed economies"[tiab] OR "under developed economy"[tiab] OR "under developed economies"[tiab] OR "underdeveloped economy"[tiab] OR "underdeveloped economies"[tiab] OR "middle income economy"[tiab] OR "middle income economies"[tiab] OR "low income economy"[tiab] OR "low income economies"[tiab] OR "lower income economy"[tiab] OR "lower income economies"[tiab] OR "low gdp"[tiab] OR "low gnp"[tiab] OR "low gross domestic"[tiab] OR "low gross national"[tiab] OR "lower gdp"[tiab] OR "lower gnp"[tiab] OR "lower gross domestic"[tiab] OR "lower gross national"[tiab] OR lmic[tiab] OR lmics[tiab] OR "third world"[tiab] OR "lami country"[tiab] OR "lami countries"[tiab] OR "transitional country"[tiab] OR "transitional countries"[tiab] OR Africa[tiab] OR Asia[tiab] OR Caribbean[tiab] OR West Indies[tiab] OR South America[tiab] OR Latin America[tiab] OR Central America[tiab] OR "Atlantic Islands"[tiab] OR "Commonwealth of Independent States"[tiab] OR "Pacific Islands"[tiab] OR "Indian Ocean Islands"[tiab] OR "Eastern Europe"[tiab] OR Afghanistan[tiab] OR Albania[tiab] OR Algeria[tiab] OR Angola[tiab] OR Armenia[tiab] OR Armenian[tiab] OR Azerbaijan[tiab] OR Bangladesh[tiab] OR Benin[tiab] OR Byelarus[tiab] OR Byelorussian[tiab] OR Belarus[tiab] OR Belorussian[tiab] OR Belorussia[tiab] OR Belize[tiab] OR Bhutan[tiab] OR Bolivia[tiab] OR Bosnia[tiab] OR Herzegovina[tiab] OR Hercegovina[tiab] OR Botswana[tiab] OR Brasil[tiab] OR Brazil[tiab] OR Bulgaria[tiab] OR Burkina Faso[tiab] OR Burkina Fasso[tiab] OR Upper Volta[tiab] OR Burundi[tiab] OR Urundi[tiab] OR Cambodia[tiab] OR Khmer Republic[tiab] OR Kampuchea[tiab] OR Cameroon[tiab] OR Cameroons[tiab] OR Cameron[tiab] OR Cape Verde[tiab] OR Central African Republic[tiab] OR Chad[tiab] OR China[tiab] OR Colombia[tiab] OR Comoros[tiab] OR Comoro Islands[tiab] OR Comores[tiab] OR Mayotte[tiab] OR Congo[tiab] OR Zaire[tiab] OR Costa Rica[tiab] OR Cote d'Ivoire[tiab] OR Ivory Coast[tiab] OR Cuba[tiab] OR Czechoslovakia[tiab] OR Slovakia[tiab] OR Djibouti[tiab] OR French Somaliland[tiab] OR Dominica[tiab] OR Dominican Republic[tiab] OR East Timor[tiab] OR East Timur[tiab] OR Timor Leste[tiab] OR Ecuador[tiab] OR Egypt[tiab] OR El Salvador[tiab] OR Eritrea[tiab] OR Ethiopia[tiab] OR Fiji[tiab] OR Gabon[tiab] OR Gabonese Republic[tiab] OR Gambia[tiab] OR Gaza[tiab] OR Georgia Republic[tiab] OR Georgian Republic[tiab] OR Ghana[tiab] OR Gold Coast[tiab] OR Grenada[tiab] OR Guatemala[tiab] OR Guinea[tiab] OR Guiana[tiab] OR Guyana[tiab] OR Haiti[tiab] OR Honduras[tiab] OR India[tiab] OR Maldives[tiab] OR Indonesia[tiab] OR Iran[tiab] OR Iraq[tiab] OR Jamaica[tiab] OR Jordan[tiab] OR Kazakhstan[tiab] OR Kazakh[tiab] OR Kenya[tiab] OR Kiribati[tiab] OR Korea[tiab] OR Kosovo[tiab] OR Kyrgyzstan[tiab] OR Kirghizia[tiab] OR Kyrgyz Republic[tiab] OR Kirghiz[tiab] OR Kirgizstan[tiab] OR "Lao PDR"[tiab] OR Laos[tiab] OR Lebanon[tiab] OR Lesotho[tiab] OR Basutoland[tiab] OR Liberia[tiab] OR Libya[tiab] OR Macedonia[tiab] OR Madagascar[tiab] OR Malagasy Republic[tiab] OR Malaysia[tiab] OR Malaya[tiab] OR Malay[tiab] OR Sabah[tiab] OR Sarawak[tiab] OR Malawi[tiab] OR Nyasaland[tiab] OR Mali[tiab] OR Marshall Islands[tiab] OR Mauritania[tiab] OR Mauritius[tiab] OR Agalega Islands[tiab] OR "Melanesia"[tiab] OR Mexico[tiab] OR Micronesia[tiab] OR Middle East[tiab] OR Moldova[tiab] OR Moldovia[tiab] OR Moldovian[tiab] OR Mongolia[tiab] OR Montenegro[tiab] OR Morocco[tiab] OR Ifni[tiab] OR Mozambique[tiab] OR Myanmar[tiab] OR Myanma[tiab] OR Burma[tiab] OR Namibia[tiab] OR Nepal[tiab] OR Nicaragua[tiab] OR Niger[tiab] OR Nigeria[tiab] OR Muscat[tiab] OR Pakistan[tiab] OR Palau[tiab] OR Palestine[tiab] OR Panama[tiab] OR Paraguay[tiab] OR Peru[tiab] OR Philippines[tiab] OR Philipines[tiab] OR Phillipines[tiab] OR Phillippines[tiab] OR Romania[tiab] OR Rumania[tiab] OR Roumania[tiab] OR Rwanda[tiab] OR Ruanda[tiab] OR Saint Kitts[tiab] OR St Kitts[tiab] OR Nevis[tiab] OR Saint Lucia[tiab] OR St Lucia[tiab] OR Saint Vincent[tiab] OR St Vincent[tiab] OR Grenadines[tiab] OR Samoa[tiab] OR Samoan Islands[tiab] OR Navigator Island[tiab] OR Navigator Islands[tiab] OR Sao Tome[tiab] OR Senegal[tiab] OR Serbia[tiab] OR Montenegro[tiab] OR Sierra Leone[tiab] OR Sri Lanka[tiab] OR Ceylon[tiab] OR Solomon Islands[tiab] OR Somalia[tiab] OR Sudan[tiab] OR Suriname[tiab] OR Surinam[tiab] OR Swaziland[tiab] OR Syria[tiab] OR Syrian[tiab] OR Tajikistan[tiab] OR Tadzhikistan[tiab] OR Tadjikistan[tiab] OR Tadzhik[tiab] OR Tanzania[tiab] OR Thailand[tiab] OR Togo[tiab] OR Togolese Republic[tiab] OR Tonga[tiab] OR Tunisia[tiab] OR Turkey[tiab] OR Turkmenistan[tiab] OR Turkmen[tiab] OR Tuvalu[tiab] OR Uganda[tiab] OR Ukraine[tiab] OR Uzbekistan[tiab] OR Uzbek OR Vanuatu[tiab] OR New Hebrides[tiab] OR Vietnam[tiab] OR Viet Nam[tiab] OR West Bank[tiab] OR Yemen[tiab] OR Yugoslavia[tiab] OR Zambia[tiab] OR Zimbabwe[tiab] OR Rhodesia[tiab] OR Developing Countries[Mesh] OR Africa[Mesh:NoExp] OR Africa, Northern[Mesh:NoExp] OR Africa South of the Sahara[Mesh:NoExp] OR Africa, Central[Mesh:NoExp] OR Africa, Eastern[Mesh:NoExp] OR Africa, Southern[Mesh:NoExp] OR Africa, Western[Mesh:NoExp] OR Asia[Mesh:NoExp] OR Asia, Central[Mesh:NoExp] OR Asia, Southeastern[Mesh:NoExp] OR Asia, Western[Mesh:NoExp] OR Caribbean Region[Mesh:NoExp] OR West Indies[Mesh:NoExp] OR South America[Mesh:NoExp] OR Latin America[Mesh:NoExp] OR Central America[Mesh:NoExp] OR "Atlantic Islands"[Mesh:NoExp] OR "Commonwealth of Independent States"[Mesh:NoExp] OR "Pacific Islands"[Mesh:NoExp] OR "Indian Ocean Islands"[Mesh:NoExp] OR "Europe, Eastern"[Mesh:NoExp] OR Afghanistan[Mesh] OR Albania[Mesh] OR Algeria[Mesh] OR American Samoa[Mesh] OR Angola[Mesh] OR Armenia[Mesh] OR Azerbaijan[Mesh] OR "Baltic States"[Mesh] OR Bangladesh[Mesh] OR Benin[Mesh] OR "Republic of Belarus"[Mesh] OR Belize[Mesh] OR Bhutan[Mesh] OR Bolivia[Mesh] OR Bosnia-Herzegovina[Mesh] OR Botswana[Mesh] OR Brazil[Mesh] OR Bulgaria[Mesh] OR Burkina Faso[Mesh] OR Burundi[Mesh] OR Cambodia[Mesh] OR Cameroon[Mesh] OR Cape Verde[Mesh] OR Central African Republic[Mesh] OR Chad[Mesh]] OR China[Mesh] OR Colombia[Mesh] OR Comoros[Mesh] OR Congo[Mesh] OR Costa Rica[Mesh] OR Cote d'Ivoire[Mesh] OR Cuba[Mesh] OR Czechoslovakia[Mesh] OR Slovakia[Mesh] OR Djibouti[Mesh] OR "Democratic Republic of the Congo"[Mesh] OR "Democratic People's Republic of Korea"[Mesh] OR Dominica[Mesh] OR Dominican Republic[Mesh] OR East Timor[Mesh] OR Ecuador[Mesh] OR Egypt[Mesh] OR El Salvador[Mesh] OR Eritrea[Mesh] OR Ethiopia[Mesh] OR Fiji[Mesh] OR "French Guiana"[Mesh] OR Gabon[Mesh] OR Gambia[Mesh] OR "Georgia (Republic)"[Mesh] OR Ghana[Mesh] OR Grenada[Mesh] OR Guatemala[Mesh] OR Guinea[Mesh] OR Guinea-Bissau[Mesh] OR Guyana[Mesh] OR Haiti[Mesh] OR Honduras[Mesh] OR "Independent State of Samoa"[Mesh] OR India[Mesh] OR Indonesia[Mesh] OR Iran[Mesh] OR Iraq[Mesh] OR Jamaica[Mesh] OR Jordan[Mesh] OR Kazakhstan[Mesh] OR Kenya[Mesh] OR Korea[Mesh] OR Kyrgyzstan[Mesh] OR Laos[Mesh] OR Lebanon[Mesh] OR Lesotho[Mesh] OR Liberia[Mesh] OR Libya[Mesh] OR "Macedonia (Republic)"[Mesh] OR Madagascar[Mesh] OR Malawi[Mesh] OR Malaysia[Mesh] OR Mali[Mesh] OR Mauritania[Mesh] OR Mauritius[Mesh] OR "Melanesia"[Mesh] OR Mexico[Mesh] OR Micronesia[Mesh] OR Middle East[Mesh:NoExp] OR Moldova[Mesh] OR Mongolia[Mesh] OR Montenegro[Mesh] OR Morocco[Mesh] OR Mozambique[Mesh] OR Myanmar[Mesh] OR Namibia[Mesh] OR Nepal[Mesh] OR Nicaragua[Mesh] OR Niger[Mesh] OR Nigeria[Mesh] OR Pakistan[Mesh] OR Palau[Mesh] OR Panama[Mesh] OR Papua New Guinea[Mesh] OR Paraguay[Mesh] OR Peru[Mesh] OR Philippines[Mesh] OR "Republic of Korea"[Mesh] OR Romania[Mesh] OR Rwanda[Mesh] OR Saint Lucia[Mesh] OR "Saint Vincent and the Grenadines"[Mesh] OR Samoa[Mesh] OR Senegal[Mesh] OR Serbia[Mesh] OR Montenegro[Mesh] OR Sierra Leone[Mesh] OR Sri Lanka[Mesh] OR Somalia[Mesh] OR South Africa[Mesh] OR Sudan[Mesh] OR Suriname[Mesh] OR Swaziland[Mesh] OR Syria[Mesh] OR Tajikistan[Mesh] OR Tanzania[Mesh] OR Thailand[Mesh] OR Togo[Mesh] OR Tonga[Mesh] OR Tunisia[Mesh] OR Turkey[Mesh] OR Turkmenistan[Mesh] OR Uganda[Mesh] OR Ukraine[Mesh] OR Uzbekistan[Mesh] OR Vanuatu[Mesh] OR Vietnam[Mesh] OR Yemen[Mesh] OR Yugoslavia[Mesh] OR Zambia[Mesh] OR Zimbabwe[Mesh] OR “Southern African Development Community”[all fields] OR “East African Community"[all fields] OR “West African Health Organisation"[all fields] OR “Sub Saharan Africa "[all fields] OR “SubSaharan Africa "[all fields])
