# Supplementary material for: Identifying health policy and systems research priorities for the sustainable development goals: social protection for health
Source: Int J Equity Health. 2018 Sep 27;17:155. doi: 10.1186/s12939-018-0868-z (PMC6161373; doi:10.1186/s12939-018-0868-z)
Supplement: Supplementary file 2 — Final research questions with original sources. (DOCX 90 kb) [file 12939_2018_868_MOESM2_ESM.docx]

**Annex** **2**: Final research questions with original sources

| **Final Question** | **Original Question** | **# Generations** | **Review Sources** | **# Policy Maker Consultations** |
| --- | --- | --- | --- | --- |
| 1. What are the effects of conditional cash transfer programs on healthcare quality, coverage and outcomes across settings in low and middle income countries? | What is the effect of conditional cash transfer programs on general mortality and health indicators across contexts and settings? | 14 | [9, 16, 18, 20, 24, 28, 34, 35] | 4: Liberia, Pakistan, PAHO, World Bank |
| 1. How do the characteristics of cash transfers such as the amount, frequency, method of payment, etc., affect intended outcomes, particularly enrollment of the target population? | How do the characteristics of cash transfers such as the amount, frequency, method of payment, affect intended outcomes? | 12 | [13, 15, 30, 44] | 0 |
| 1. What are the impacts of social protection programs in conflict affected settings and their effectiveness in improving health outcomes and access to health services? | How effective are social protection programs in conflict affected settings in improving health outcomes and access to health services? | 9 | [22] | 2: Somalia, Liberia |
| 1. What are the long-term effects of cash transfer programs or social protection programs on behavior change, and how sustainable have they been? | What are the long-term effects and sustainability of health behavior change that have been affected through cash transfer programs or social protection programs? | 7 | [13, 25, 27, 43] | 5: Indonesia (x2), South Africa, PAHO, World Bank |
| 1. How can demand-side financing for health be made financially sustainable? | What is the financial sustainability of demand-side financing for health? | 12 | [13, 27, 34] | 0 |
| 1. How can social protection programs for health be designed, implemented, and evaluated to ensure sustainability and scalability in low and middle income countries, including conflict affected settings? | How can social protection programs for health be designed and implemented to ensure sustainability and scalability in low and middle income countries, including conflict affected settings? | 5 | [13, 22, 34] | 2: Liberia, Pakistan |
| 1. What is the impact of social protection initiatives on health equity outcomes and equitable access to quality health care services for poor and marginalized populations? | What are the impacts of social protection initiatives on equity and equitable access to health care services for the most poor and marginalized populations? | 10 | [17, 26, 31, 34] | 3: Jordan, Indonesia, South Africa |
| 1. How do the characteristics of food aid programs (e.g. source, amount, frequency, mode, recipient etc.) affect intended health-related outcomes (e.g. morbidity, sustained behavior changes, drug adherence, labor market participation etc.)? | How do the characteristics of food aid programs (e.g. amount, frequency, mode, etc.) affect intended health-related outcomes (e.g. morbidity, sustained behavior changes, drug adherence, labor market participation etc.)? | 5 | [23, 42, 43] | 0 |
| 1. What are the impacts of different forms of maternity leave (paid vs. unpaid, length, etc.) on maternal and child health outcomes? | How does maternity leave affect maternal health outcomes? | 12 | [44] | 0 |
| 1. How do demand-side financing or CCT programs affect out-of-pocket spending on health? | How do demand-side financing or CCT programs affect out of pocket spending on health? | 9 | [12] | 1: Kenya |
| 1. How cost-effective are CCT programs compared to supply-side interventions (e.g. strengthening quality of infrastructure and expanding services) in improving health? | What is the cost-effectiveness of CCT programs compared with supply-side interventions (e.g. strengthening quality of infrastructure and expanding services) in improving health? | 7 | [15]  [27, 31] | 0 |
| 1. How cost-effective are CCT programs compared with other types of demand side interventions (e.g. UCTs, vouchers, behavior change, communication) in improving health coverage and health outcomes? | What is the effectiveness and cost-effectiveness of CCT programs compared with other types of demand side interventions (e.g. UCTs, vouchers, behavior change, communication) in improving health? | 7 | [13, 25, 31, 34, 37, 39] | 1: Tunisia |
| 1. What are the pathways through which social protection programs affect clinical and nonclinical outcomes, and what are the implications on program design? | What are the pathways through which social protection programs affect clinical and non-clinical outcomes, and what are the implications for program design? | 9 | [12, 13, 15, 18, 25, 28, 30, 31, 33, 34, 42, 44] | 0 |
| 1. How do social protection programs (e.g. cash transfers) affect intergenerational and gender relationships at the community and household level? | How do particular social protection initiatives (e.g. cash transfers) affect intergenerational and gender relationships at the community and household level? | 6 | [30, 43] | 1: World Bank |
| 1. How does conditionality in cash transfer programs affect behavioral changes for disease prevention and treatment? | How important is conditionality in cash transfer programs to achieving the desired behavioral changes for disease prevention and treatment? | 6 | [13, 19, 34] | 0 |
| 1. What are the unintended health-related consequences of social protection programs? | What are the unintentional health-related consequences of social protection programs? | 5 | [13, 19] | 3: India, Tunisia, World Bank |
| 1. How can social protection programs be designed to minimize dependency and promote productivity amongst beneficiaries? | How can social protection programs be designed to minimize dependency and promote productivity amongst beneficiaries? | 7 | [43] | 3: South Africa, Jordan (x2) |
| 1. What is the extent of fraud and abuse in health-related social protection programs, and how can social protection programs be designed to show accountability? | To what extent is there fraud and abuse in health-related social protection programs, and how can social protection programs be designed to minimize this? | 8 | 0 | 6: Philippines (x2), India, South Africa, Bahrain, Jordan |
| 1. What tools and systems can be used to assess and apply eligibility criteria for health-related social protection programs? | What tools and systems can be implemented to determine eligibility for health-related social protection programs? | 9 | [29, 34, 43] | 8: Indonesia (x2), Ghana, Laos, IADB, India, Jordan, Bahrain |
| 1. How can the community/civil society be engaged to help design, implement and evaluate social protection programs? | How can the community/civil society be included in the design and implementation of social protection programs? | 6 | [27, 32] | 2: Argentina, Indonesia |
| 1. How can various social protection initiatives be best integrated or harmonized across sectors? | How can we best integrate or harmonize various social protection initiatives across sectors? | 6 | [13] | 12: Laos, South Africa, India (x2), Indonesia, Argentina (x2), IADB, R4D, WB, Kiribati, Myanmar |
| 1. How do social protection programs influence the interaction between public and private health care providers with regards to service availability, quality of care and utilization? | How do social protection programs influence the interaction between public and private health care providers, and in turn, availability of services and quality of care? | 7 | [22, 27] | 1: India |
| 1. How do social protection programs contribute to state building? | Does the provision of social protection programs contribute to state building? | 6 | [22] | 0 |
| 1. How do we provide social protection programs to refugee populations without undermining support for nationals? | How do we provide social protection programs to refugee populations without undermining support for nationals? | 6 | [22] | 1: Jordan |
| 1. How can routine information systems be strengthened and used to monitor and evaluate social protection systems for health? | How can routine information systems be strengthened so as to facilitate assessment of the use and effect of social protection systems on health? | 6 | [38] | 13: Indonesia, ADB, Philippines, India (x5), South Africa (x2), Liberia (x2), R4D, |
| 1. How can informal sector and migrant workers be effectively covered by health-related social protection systems? | How can informal sector & migrant workers best be integrated in health-related social protection systems? | 10 | 0 | 5: Indonesia, R4D, WHO, WB (x2) |
| 1. How can social protection systems help in protecting people from domestic violence and its consequences? | What role can social protection systems play in protecting people from domestic violence and its consequences? | 8 | 0 | 3: South Africa, Vanuatu, Jordan |
| 1. How can social protection schemes help in ensuring that the most vulnerable such as the disabled are provided with people-centered and integrated services? | What is the role of social protection schemes in ensuring people-centered and integrated services for the most vulnerable people in society such as the disabled? | 6 | [29, 32] | 1: Jordan |
| 1. What are the contextual factors that influence the effectiveness of conditional and unconditional cash transfer schemes for health? | How does context influence the effectiveness of conditional and unconditional cash transfer schemes for health? | 7 | [15, 39] | 3: Bhutan, Vanuatu, China |
| 1. What are the effects of unconditional cash transfer programs on healthcare quality, coverage and outcomes across settings in low and middle income countries? | What is the effect of unconditional cash transfer programs on general mortality and health indicators across contexts and settings? | 8 | [9, 16, 18, 20, 27, 34, 35] | 3: Liberia, PAHO, World Bank |
| 1. How do social protection programs for health affect intergenerational poverty and social mobility? | **N/A- new question generated after Round 1** | N/A |  |  |
